# CAT-CBAM-Net: An Automatic Scoring Method for Sow Body Condition Based on CNN and Transformer

**DOI:** 10.3390/s23187919

**Published:** 2023-09-15

**Authors:** Hongxiang Xue, Yuwen Sun, Jinxin Chen, Haonan Tian, Zihao Liu, Mingxia Shen, Longshen Liu

**Affiliations:** 1 College of Engineering, Nanjing Agricultural University, Nanjing 210031, China; 2020212014@stu.njau.edu.cn (H.X.); syw-ln@njau.edu.cn (Y.S.); chenjinxin@stu.njau.edu.cn (J.C.); 9203020113@stu.njau.edu.cn (Z.L.); 2 Key Laboratory of Breeding Equipment, Ministry of Agriculture and Rural Affairs, Nanjing 210031, China; 9203021221@stu.njau.edu.cn (H.T.); mingxia@njau.edu.cn (M.S.); 3 College of Artificial Intelligence, Nanjing Agricultural University, Nanjing 210031, China

**Keywords:** parturient sows, artificial neural networks, body condition score, machine vision

## Abstract

Sow body condition scoring has been confirmed as a vital procedure in sow management. A timely and accurate assessment of the body condition of a sow is conducive to determining nutritional supply, and it takes on critical significance in enhancing sow reproductive performance. Manual sow body condition scoring methods have been extensively employed in large-scale sow farms, which are time-consuming and labor-intensive. To address the above-mentioned problem, a dual neural network-based automatic scoring method was developed in this study for sow body condition. The developed method aims to enhance the ability to capture local features and global information in sow images by combining CNN and transformer networks. Moreover, it introduces a CBAM module to help the network pay more attention to crucial feature channels while suppressing attention to irrelevant channels. To tackle the problem of imbalanced categories and mislabeling of body condition data, the original loss function was substituted with the optimized focal loss function. As indicated by the model test, the sow body condition classification achieved an average precision of 91.06%, the average recall rate was 91.58%, and the average F1 score reached 91.31%. The comprehensive comparative experimental results suggested that the proposed method yielded optimal performance on this dataset. The method developed in this study is capable of achieving automatic scoring of sow body condition, and it shows broad and promising applications.

## 1. Introduction

Body condition scoring (BCS) refers to an essential link existing in the management of multiparous sows. The reproductive performance and longevity of multiparous sows can be enhanced through timely assessment of their body condition and adjustment of their feeding level [1]. In general, the scoring of sow body condition conforms to manual visual inspection on current large-scale sow farms. The above-described method is limited by the scoring experience of technicians, and it can easily cause stress reactions in sows. The analysis of the above method reveals that it is challenging to satisfy the requirements of large-scale breeding for scoring sow body condition [2].

As the sow breeding scale has been rising continuously, automated and intelligent management of sows should be urgently performed. Research on non-contact automatic scoring methods for sow body condition has progressively become a research hotspot, which primarily covers two types of methods based on 2D image processing and 3D image reconstruction. Teng et al. [3] captured 3D images of sow buttocks using handheld Kinect cameras and achieved non-contact detection of sow body condition using 3D reconstruction technology. Although the 3D image is capable of obtaining the degree of bone exposure, body concavity, and convex degree indicating the sow’s body condition, the Kinect camera has a higher cost and more rigorous shooting requirements; the capturing of 3D data turns out to be more sophisticated; and the cost of 2D image acquisition equipment and data requirements are lower. Accordingly, the development of a sow body condition scoring system based on 2D images displays pronounced advantages in application.

Huang et al. [4] investigated the scoring of sow body condition based on 2D. They obtained the height–width ratio and curvature of the sow hip through image segmentation, ellipse fitting, and linear regression. The height–width ratio and curvature of the sow hip considerably indicated the sow body condition, and the use of 2D images generated the sow body condition score. Yuan [5] obtained the main descriptive characteristics of hip morphology using image segmentation models and statistical analysis methods. In brief, most of the existing scoring methods for sow body condition require images of sows, and the processing is complicated, which cannot conform to the automatic scoring requirements for the practical breeding condition of a sow body.

In this study, a scoring method for sow body condition was proposed based on the CAT-CBAM-Net neural network in accordance with the collected image data and body condition scoring data to further facilitate the automation of the scoring of sow body condition. The main contributions of this study are presented as follows:

(1) The score data for the sow body condition were processed, and the score dataset for the sow body condition was established.
(2) A body condition scoring model was built based on the CAT-CBAM-Net hybrid network by employing transfer learning, network fusion, and loss function optimization to reconstruct the model.
(3) For practical breeding, experiments validated the good performance of the developed method for scoring sow body condition. This method takes on great significance in promoting automation, increasing the accuracy of sow body condition scoring methods, and enhancing sow reproductive performance.

## 2. Materials and Methods

### 2.1. Experimental Data Collection

The images of multiparous sows were captured on a large-scale farm in Yancheng City, Jiangsu Province, China, from 15 September 2021 to 5 August 2022. 5000 sows (crossbred sows bred by Yorkshire boars and Landrace sows) were selected as the experimental objects of this study. The experimental sows were multiparous sows of the same age with parity 2. The data were collected from sows during empty pregnancy, estrus, and pregnancy (pre-pregnancy, mid-pregnancy, and late-pregnancy). The sows data collection was performed every day after feeding. During the experiment, three expert veterinarians scored their body conditions once, and the scoring was supported by using a sow caliper (YORK, BBKC, Jinan, China). All experimental designs and procedures of this study gained approval from the Committee of Animal Research Institute, Nanjing Agricultural University, Nanjing, China (Certification NJAU.No20210906086).

**Figure 1:**
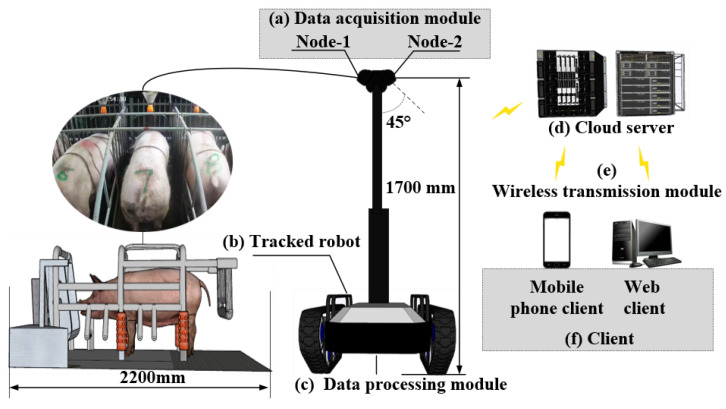
Collection and processing system for sow body condition scoring data. (**a**) A data acquisition module composed of two data acquisition nodes. (**b**) Tracked robot. (**c**) Data processing module for data analysis and frame truncation. (**d**) Cloud server for model deployment and data storage. (**e**) Wireless transmission module for real-time data transmission between different devices. (**f**) Client for real-time display of information.

In general, the data acquisition and processing system was comprised of the tracked robot, the data acquisition module, and the data processing module (Figure 1). The data acquisition module was comprised of two data acquisition nodes (HIKVISION, DS-T12HV3-IA, Hangzhou, China), with a resolution of 1920 × 1080 pixels. To be specific, the nodes were fixed on a bar with the top 1700 mm above the ground and a 45° downward vertical angle. The images were uploaded to the cloud servers based on the wireless transmission module. Furthermore, the images of the sow’s body condition could be downloaded from the cloud servers to the local server via the application programming interface.

### 2.2. Dataset Preparation

The preparation of the experimental dataset included data formatting, data cleaning, and data augmentation.

#### 2.2.1. Dataset Formatting

Different sow objects existed in an image; thus, the classification results of the target sows would be considerably perturbed. The original image was clipped to ensure the accuracy of the classification results. The image was trimmed as a rectangular box with coordinates of (500, 0), (1250, 0), (0, 1080), and (500, 1080). Subsequently, the images were divided into training, validation, and test sets in a ratio of 8:1:1. The training set was used to train the BCS model; the validation set was used to adjust the parameters and select the optimal model; and the test set was used to assess the performance of the sow BCS model [6].

#### 2.2.2. Data Cleaning and Augmentation

**Figure 2:**
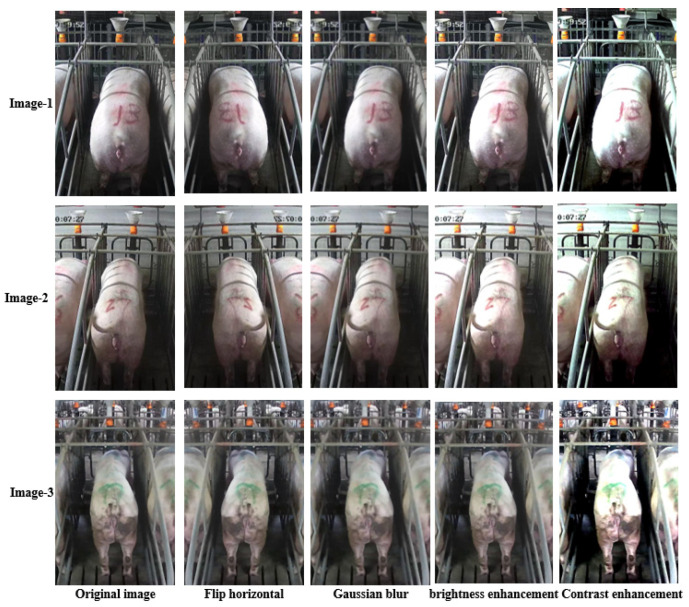
Original and enhanced images.

Images with inadequate exposure and high similarity were eliminated after obtaining images of the sow BCS. Data augmentation methods (horizontal flipping, additive Gaussian noise and brightness adjustment, etc.) were used to amplify the dataset to enrich the data representation and improve the generalization performance of the model [7]. The original 4938 images were expanded to 6000. The results of different image expansion methods are shown in Figure 2. The sow scoring results were mapped to the amplified image, and the sows were divided into 5 grades according to their degrees of obesity (Table 1) [8].

### 2.3. Establishment of the Sow BCS Model

**Figure 3:**
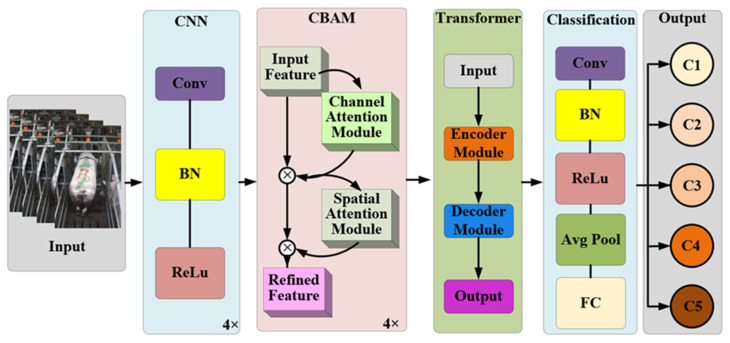
Model framework.

In sow production, manual BCS requires a combination of the overall body size and key assessment points (e.g., the spine, hip bone, and tail root). Likewise, the local and global features of the sow images should be extracted when machine vision is adopted to classify the sow’s body condition. The convolutional neural network (CNN) exhibits prominent local feature extraction capability, whereas it is subjected to a limited perceptual field and poor global representation capability [9]. In addition, the visual transformer covers a self-attention network, a cross-attention network, and position-encoding modules, which have certain global information interaction capabilities but poor local feature capture capabilities [10]. The CNN fused with the visual transformer is capable of capturing local and global information from sow images; Figure 3 depicts the framework of the built model.

As depicted in Figure 3, the sow BCS model principally involved the CNN feature extraction, CBAM, transformer, and classification modules. To be specific, the CNN feature extraction module extracts local features hierarchically through convolutional operations as feature maps. The convolutional block attention module (CBAM) enables the model to focus on essential feature channels via the joint channel attention and spatial attention modules. The transformer module compensates for the global feature extraction of CNN through cascaded self-attention modules. Furthermore, the classification module fuses the output from the aforementioned modules and maps the feature dimensions to the number of categories.

#### 2.3.1. CNN Feature Extraction Module

**Figure 4:**
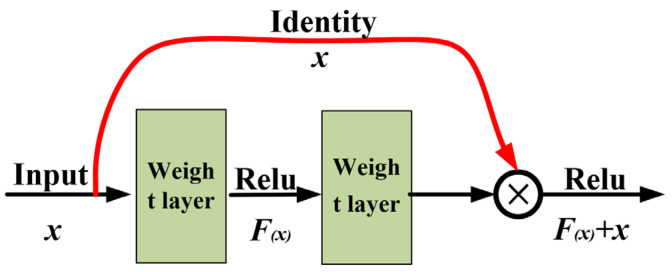
Residual structure.

Skeletal assessment points are relevant detailed features of sow body condition scores; the accurate capture of information on the above-mentioned local features assists the model to classify sow images. ResNet-18 serves as the backbone network and is compared with the CNN network. ResNet-18 introduces a residual structure, which can increase the efficiency of information propagation by skipping connections [11,12]. Figure 4 illustrates the residual structure, and the residual structure satisfies Equation (1) [13].

(1) $$x_{i+1}=f[x_{i}+F(x_{i},k_{m})]$$

where $x_{i}$ denotes the input of the ith residual structure, $x_{i+1}$ represents the output of the ith residual structure, *f* expresses the activation function, *F* is the residual function, and *k* is the convolution kernel [14].

#### 2.3.2. CBAM

**Figure 5:**
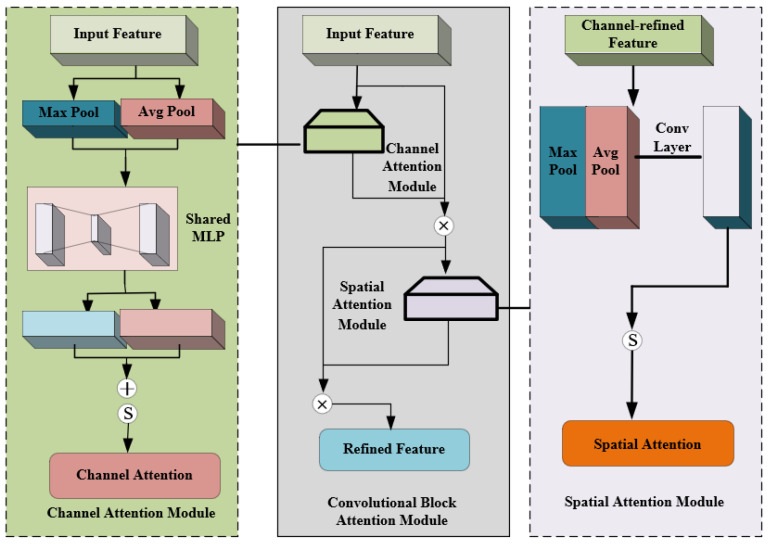
CBAM.

CBAM was introduced, which was aimed at enabling the model to focus on relevant feature channels and suppress irrelevant feature channels [15]. CBAM refers to a simple and effective feed-forward CNN that is capable of inferring the attention map along the channel attention module and spatial attention module for a given feature map and multiplying the input feature map with the attention map for feature optimization [16,17] (Figure 5).

#### 2.3.3. Transformer Module

**Figure 6:**
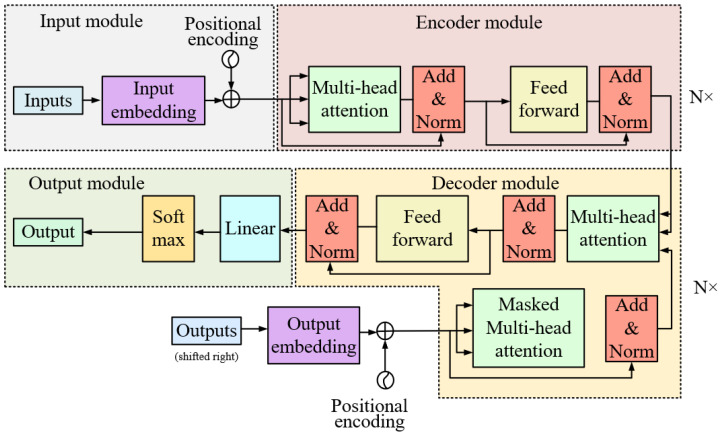
Transformer model architecture diagram.

Transformer refers to an attention mechanism-based network model architecture. It has been first applied in natural language processing and then extensively employed in computer vision [18]. As depicted in Figure 6, the transformer model covers four main parts, i.e., the input module, the encoding module, the decoding module, and the output module.

The first part refers to the input module, which is comprised of two parts, i.e., the input embedding module and the positional encoding module. To be specific, the input embedding module is adopted to convert the vocabulary in the text sequence into a vector representation and capture the semantic relationship of the vocabulary. Moreover, the positional encoding module is employed to introduce positional information to the vector representation of the respective vocabulary, enabling the model to process the order and position relationships in the input sequence. The combination of the two enables the transformer to gain more insight into and process the semantics and structure of the input sequence and to enable modeling capabilities for long-distance dependencies [19].

The encoding module acts as the second part, and its function is to extract features and model context information. It principally covers the multi-head attention layer, the feed-forward neural network, residual connections, and layer normalization. The multi-head attention layer captures different semantic information in the input sequence by computing multiple attention heads in parallel. The feed-forward neural network applies nonlinear transformations and mappings to the output of the multi-head attention layer. The inputs of the multi-head attention layer and the feed-forward neural network are combined with the outputs of their respective sub-modules through residual connections, with the aim of more effectively propagating information, alleviating the problem of gradient vanishing, and accelerating the training process [20].

The decoding module acts as the third part, which plays a role in generating the target sequence. The decoding module is stacked by N decoders, and the N value conforms to the N value of the encoding module. The decoder falls into three layers. The first sublayer receives the previous output of the decoder stack. Consequently, this output is augmented by the first sublayer with positional information, and multi-head self-attention is implemented on the output. The second layer implements the self-attention mechanism of bulls. On the decoder side, this multi-head mechanism receives queries from the previous decoder sublayer as well as keys and values from the encoder output. Furthermore, a fully connected feed-forward network is implemented by the third layer [21].

The fourth part refers to the output module; the output part contains two parts: linearization and softmax regression. Linearization maps the vector output of the decoder structure to a longer vector; each number in the vector represents the score of one vocabulary. Softmax regression is used to convert the above-mentioned scores into the probability of the words in the corresponding dictionary and finally select the word with the highest probability as the current output [22].

#### 2.3.4. Classification Module

The classification module is comprised of five main parts, i.e., a convolutional layer, a batch normalization layer, a rectified linear unit, an average pooling layer, and a fully connected layer. The size of the convolutional kernel is 1 × 1 to ensure that the model is computationally minimal, which ensures that the feature map size remains unchanged. A global average pooling layer was adopted to perform the averaging operation on the respective feature map to achieve feature downscaling and global picture enhancement features. The fully connected layer mapped the dimensionality to the number of categories for image classification.

#### 2.3.5. Optimization of the Loss Function

A category imbalance and mislabeling problem was identified in the sow body condition score dataset. The focal loss (FL) function is capable of resolving the category imbalance problem, whereas it cannot address the mislabeling problem [23]. In this study, the FL function was optimized to remove mislabeled sow data and enhance the sow body condition classification performance, which is expressed by Equation (2).

(2) $$\left\{\begin{array}{c} p_{\mathrm{top}}=\varepsilon (p_{top}>c\;\&\;y_{p}\neq y_{t})\\ -\alpha {\left(1-p_{i}\right)}^{\gamma }\mathrm{lg}(p_{i})\;(other) \end{array}\right.$$

where $P_{top}$ denotes the maximum confidence of the five categories of sow body condition score images, *ε* represents the minimum value, and *c* expresses the probability threshold. $y_{P}$ denotes the predicted label of the sample, $y_{t}$ is the true label of the sample, *α* is the balance parameter, *P*_i_ is the confidence level of each category of body condition score samples, and γ represents the focus parameter. ${\left(1-P_{i}\right)}^{\gamma }$ expresses the modulation coefficient, which is used to increase the attention of the network to the hard-to-learn samples. The loss function balances the number of samples between each category by balancing the parameter. When the predicted label of a sample does not equal its true label, the sample turns out to be a mislabeled sample with high confidence, its predicted probability is set at a minimal value *ε*, and the mislabeled image is removed from the study sample.

### 2.4. Model Training

The model was dependent on the PyTorch 1.7.1 deep learning library, which conformed to the 11th Intel^®^ CoreTM i7-11700k@2.50 HZ×16 (manufactured by Intel Corp., Santa Clara, CA, USA); the graphics card was NVIDIA GTX3090 (manufactured by NVIDIA Corp., Santa Clara, CA, USA); the graphics memory was 24G; and the 64-bit Ubuntu 20.04.1LTS operating system was configured with Python 3.8, Cuda11.4, and OpenCv4.5.1.

The learning rate was dynamically adjusted by employing the cosine annealing scheduler with a burn-in of 2000 epochs so as to expedite the model convergence. The parameters were recorded every 50th epoch, the patch size was set at 32, the learning rate was set at 0.01, the batch size was set at 32, and the decay rate of weights was set at 0.05. Pre-training was performed based on the ImageNet dataset to enhance the performance of the model before the sow body condition score data were imported into the network.

### 2.5. Model Assessment

The assessment indexes of the model applied covered model size, parameter size, floating-point operations (FLOPs), Top-1 accuracy, precision (*P*, given by Equation (4)), recall (*R* computed by Equation (5)), and *F*_1_ score (*F*_1,_ obtained by Equation (6)) [24]. The spatial complexity of the model increased with an increase in the number of parameters. *F*_1_ denotes the harmonic average of precision and recall.

(3) $$A=\frac{f_{\mathrm{TP}}+f_{\mathrm{TN}}}{f_{\mathrm{TP}}+f_{\mathrm{TN}}+f_{\mathrm{FP}}+f_{\mathrm{FN}}}$$

(4) $$P=\frac{f_{\mathrm{TP}}}{f_{\mathrm{TP}}+f_{\mathrm{FP}}}$$

(5) $$R=\frac{f_{\mathrm{TP}}}{f_{\mathrm{TP}}+f_{\mathrm{FN}}}$$

(6) $$F_{1}=2\frac{P\times R}{P+R}$$

where *A* denotes the accuracy; Top-1 accuracy represents the fact that the correct classification is among the top 1 predictions output by the model. *f*_TP_ expresses the number of true positives, *f*_TN_ denotes the number of true negatives, *f*_FP_ is the number of false positives, and *f*_FN_ represents the number of false negatives.

## 3. Results

### 3.1. Model Training

**Figure 7:**
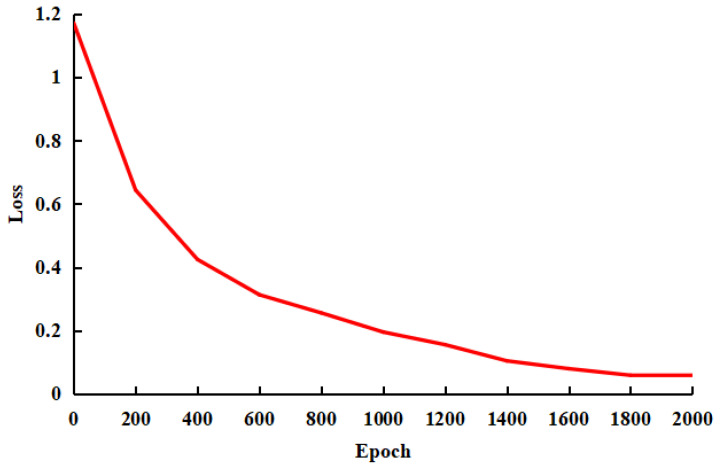
Loss curve of the model.

**Figure 8:**
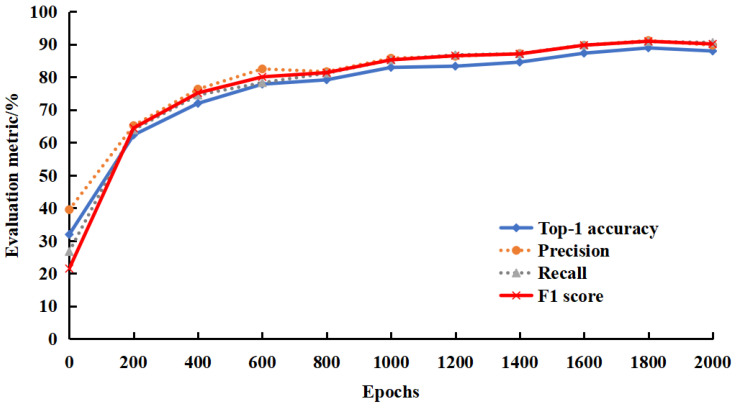
Assessment metric curve.

As depicted in Figure 7, the loss of the model tends to decrease with the rise of the training epochs. In the last 50 epochs, the loss value varied by only 0.013, and the model tended to converge. As depicted in Figure 8, the mean precision, recall, and *F*1 score of the model increased continuously over 2000 epochs and tended to stabilize. In the final 50 epochs, the loss only declined by 0.81%, the Top-1 accuracy varied by 0.38%, the mean precision changed by 0.71%, the mean recall varied by 0.68%, the mean *F*1 score changed by 0.67%, and the model converged.

**Figure 9:**
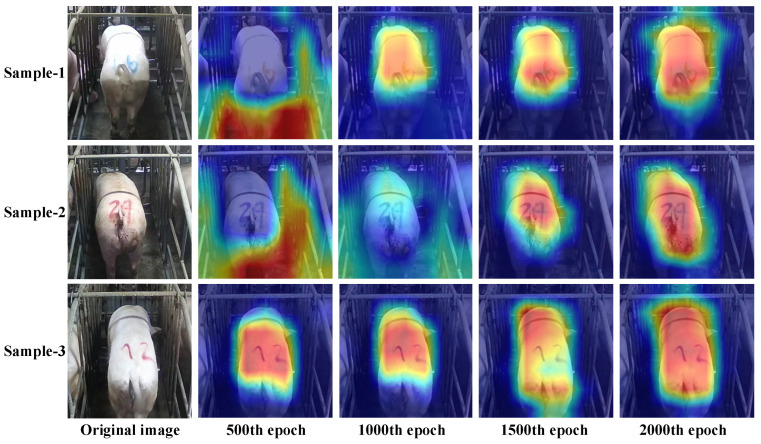
Class activation map of different epochs.

A range of sow images were examined and analyzed by employing a class activation map visualization method. Next, the examination and analysis results were adopted to assess the feature extraction ability exhibited by the CAT-CBAM-Net model at different epochs. The activation maps were smoothed through principal component analysis. The final attention module of the end layer was the target layer of the visualization. Figure 9 presents the test results. The more significant red region represents the higher attention paid by the model to this region. The model placed a greater focus on the back and rump regions of the sow with an increase in epochs, which is consistent with the attention of manual scoring.

### 3.2. Model Testing

The trained model was used to classify the test set to assess the performance of the model; the test results are listed in Table 2, according to which the average precision, recall, and F1 score are 91.06%, 91.58%, and 91.31%, respectively, and the model performance satisfied the demand of sow management.

**Figure 10:**
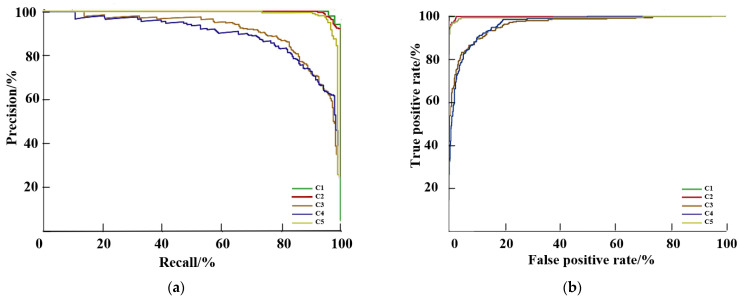
PR and ROC curves: (**a**) PR curve; (**b**) ROC curve.

The precision-recall (PR) curves and receiver operating characteristic (ROC) curves of the model are presented in Figure 10. For the PR curve, the area enclosed by the curve and coordinate axis represents the average accuracy, AP [25]. For the ROC curve, the area enclosed by the curve and the axis is the area under the curve (AUC); the performance of the classifier is considered better when the AUC is closer to 1 [26]. Based on the comparison, the model has a better classification for C1, C2, and C5, and a relatively poor classification for C3 and C4. This results from the relatively small difference between C3 and C4 sows, leading to a certain degree of misclassification.

### 3.3. Comparison of Different Loss Functions

**Figure 11:**
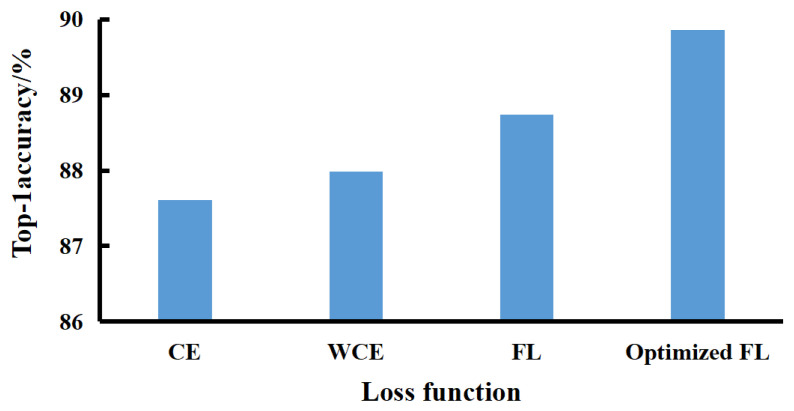
Comparison of different loss functions.

To verify the effectiveness of the optimized FL function for the model, the cross-entropy (CE) loss function, weighted cross-entropy (WCE) loss function, and FL function were selected for comparison. As depicted in Figure 11, the optimized cross-entropy loss function Top-1 accuracy has increased by 2.25%, which is 1.87% higher than the WCE loss function Top-1 accuracy and 1.82% higher than the FL function Top-1 accuracy. Therefore, the optimized FL function is helpful in solving the problems of imbalanced sow images and data mislabeling.

### 3.4. Contrast Models

The optimized model was compared with typical CNN models (e.g., EfficientNet-B0, VGG-16, ResNet-18, and AlexNet) and typical visual transformer models (e.g., ViT-base-32, T2T-ViT, and Swin-Transformer) to further validate the advantages of the CAT-CBAM-Net model in sow body condition scoring. Table 3 lists the comparison results. The AlexNet model outperformed the other CNN models for classification performance, and the Top-1 accuracy of the CAT-CBAM-Net model increased by 7.38%. Although the model size and FLOPs increased, the proposed model had fewer parameters, such that its lower spatial complexity was verified. The ViT-base-32 model outperformed the other visual transformer models for classification performance; the Top-1 accuracy of the CAT-CBAM-Net model was improved by 15.62%; and the model size and parameters were significantly reduced. The ablative experiment suggested that the CAT-CBAM-Net model, compared with the model without using the CBAM module, shows minimal increases in model size, parameter amount, and FLOPs. However, it achieved a 2.21% increase in Top-1 accuracy. The CAT-CBAM-Net model is capable of effectively extracting local and global features by integrating the CNN, CBAM, and transformer modules. Thus, the CAT-CBAM-Net model outstripped other models for sow body condition scoring.

## 4. Discussion

### 4.1. Effect of the Sow Body Condition on Its Estrus and Reproduction

For the validity of this method statement for sow production guidance, this study analyzes the relationship between body condition, estrus, and reproduction of empty sows using the proposed model as an assessment tool, combining estrus assessment indexes such as 5d estrus rate (ER-5d), 7d estrus rate (ER-7d), estrus standing rate (ESR), conception rate (CR), and repeat rate (RR) as well as reproductive assessment indexes such as average total number born (ATNB), average number born alive (ANBA), average number born healthy (ANBH), and average birth litter weight (ABLW). Twenty-five sows (C2–C5) were selected for the experiment. A total of 25 sows (C2–C5), all of which had just finished lactation, were selected for the experiment. Sows belonging to C1 consume significant amounts of body fat during lactation; therefore, the feeding strategy should be adjusted and the estrus cycle delayed before breeding sows [27,28].

**Figure 12:**
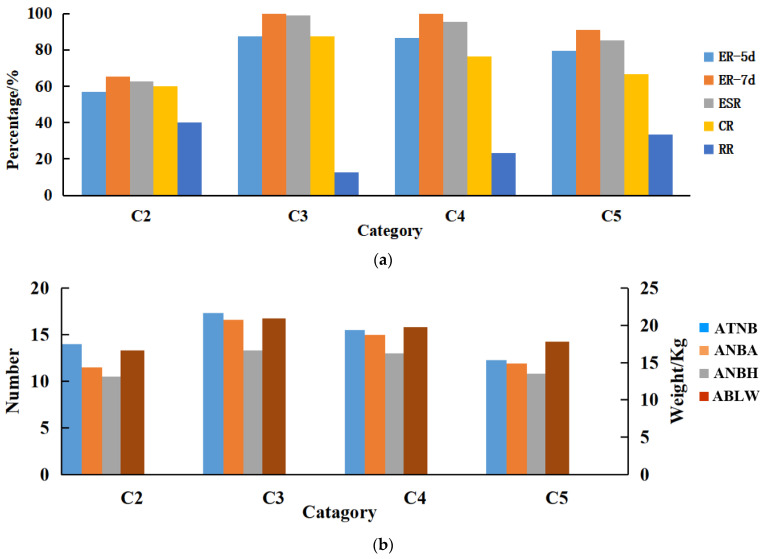
Effect of the sow body condition on its estrus and reproduction: (**a**) Effect of sow body condition on its estrus; (**b**) Effect of sow body condition on its reproductive.

As depicted in Figure 12a, the estrus performance of sows in C3 and C4 was better than that of C2 and C5, and C3 was better than C4. This result suggested that maintaining an ideal body condition can enhance the estrus performance of sows. The ER-5d, ER-7d, ESR, and CR of C5 exceeded those of C3, whereas the RR was lower than that of C3. Based on existing research, the high consumption of body fat in sows during lactation can trigger a lack of estrogen secretion, hypogonadism, silent estrus, or postpartum anestrus [29].

As depicted in Figure 12b, sows in C3 and C4 outperformed those in C2 and C5 for reproductive performance; sows in C3 outstripped those in C4, which indicated that fat and thin sows were not conducive to the improvement of reproductive performance. The ATNB of sows in C5 was lower than that of sows in C2, which suggested that the embryo survival of sows with a lean body condition was higher than that of fat sows. However, ANBA, ANBH, and ABLW were better in fat sows than those in thin sows, which suggested that fat sows can provide relatively adequate nutrition for the fetus during embryonic development. The number of sows employed for experiments was limited in the study of the estrus and reproductive performance of sows. In the subsequent research, the number of experimental sows will increase, and the mechanism of the effect of sow body condition on their estrus and reproductive performance will be analyzed in depth by combining feeding management, environmental factors, genetic factors, etc.

### 4.2. Comparison to Existing Methods

A comparison was drawn between this method and existing research to assess the superiority and practicality of this method comprehensively. The current image data of existing studies is manually acquired, and the models built using the above-mentioned data cannot be directly applied to the automatic inspection system of pig farms. In the study of a 2D-based sow body condition scoring method, a sow body condition scoring method was proposed in [4] based on semantic segmentation and feature parameter quantization. Compared with the existing method, this method is susceptible to sow posture and shooting angle. Feature parameter extraction cannot be effectively performed when there is occlusion, such as railings. In the study of 3D-based body condition scoring methods for sows, a body condition scoring method based on the radius of curvature of the sow’s rump is proposed in the literature [3]. This method rebuilds the 3D model of the sow’s hindquarters through point cloud registration, rendering techniques, and point cloud denoising. Compared with the method proposed in this study, this method is capable of capturing the 3D information of sows more comprehensively, whereas the acquisition equipment is costly and extremely inefficient. Furthermore, the model does not work properly when the sow is in motion.

### 4.3. Current Deficiencies and Subsequent Studies

Although a preliminary sow body condition scoring model was built in this study, some problems remained. As depicted in Table 2, sows in C3 and C4 were subjected to some misdetection. In the subsequent research, we will communicate with professional veterinarians to further optimize the classification method and expand the test range. Furthermore, we will work on the lightweight of the model to increase the inference speed of the model and lower the cost of deployment.

## 5. Conclusions

A CAT-CBAM-Net sow body condition scoring method was developed to address the low efficiency of manual sow body condition scoring. The body condition scoring accuracy of sows was increased by integrating the CNN, the transformer network, and the CBAM module, and the optimized FL function was introduced to tackle two problems (i.e., imbalanced body condition categories and data mislabeling). Through testing, the average precision of the model was obtained at 91.06%, with a Top-1 accuracy of 89.86%, which was higher than that of some prominent mainstream classification algorithms. The developed method can help breeders monitor sow body condition and optimize feeding management to enhance production performance and increase the reproductive efficiency of sows.

## Figures and Tables

**Table 1:** Sow body condition category.

Category	The Original Sample Size	Definition	Sample
C1	350	Rear view: extremely narrow spine angle, obvious protrusion of the tail root and hip bone; the sow is extremely thin overall.	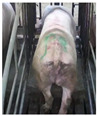
C2	947	Rear view: narrow spine angle, slight protrusion of hip bone and tail root; the sow is overall thin.	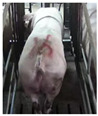
C3	1450	Rear view; normal spine angle, no protrusion at the tail root, hip bone can be touched; the sow is in ideal condition.	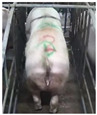
C4	1331	Rear view: wide spine angle, flat and smooth tail root, hip bone cannot be touched, and the sow is fat overall.	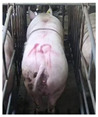
C5	860	Rear view: very wide spine angle; flat and smooth tail root; hip bone cannot be touched; the sow is overall extremely fat.	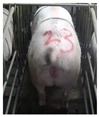

**Table 2:** Model test results.

Categories	Precision/(%)	Recall/(%)	F1 Score/(%)
C1	95.92	97.92	96.91
C2	96.31	97.03	96.67
C3	85.51	83.45	84.47
C4	82.64	82.33	82.49
C5	94.93	97.17	96.04

**Table 3:** Performance of different models.

Model	Model Size /(MB)	Parameters /(M)	FLOPs /(GFLOPs)	Top-1 Accuracy /(%)
EfficientNet-B0	33.60	4.01	0.02	79.48
VGG-16	1075.38	134.28	15.50	72.06
ResNet-18	90.40	11.18	1.82	56.16
AlexNet	436.08	57.02	0.71	82.48
ViT-base-32	1028.72	89.83	4.36	74.24
T2T-ViT	254.55	21.08	4.34	71.59
Swin-Transformer	1042.87	86.75	15.14	55.21
CAT-Net	448.58	36.43	6.89	87.65
CAT-CBAM-Net	450.20	36.48	6.90	89.86

## Data Availability

Not applicable.
